# The Principles of Physiology Applied to the Preservation of Health, and to the Improvement of Physical and Mental Education

**Published:** 1844-04

**Authors:** 


Art. X.—*The Principles of Physiology applied to the Preservation of Health, and to the Improvement of Physical and Mental Education.* By Andrew Combe, m.d. &c. With Fifteen Woodcuts. Twelfth Edition, revised and enlarged.—*Edinburgh*, 1843. Royal 8vo, Double columns, pp. 108.

On more than one occasion in this Journal, we have noticed and eulogised the admirable work, now again before us in a new edition and in an altered form. We believe that no medical work ever published, has exerted a more favorable influence, in a hygienic point of view, than this; and it affords us unmixed satisfaction to see that its truly excellent author has been able, under the pressure of prolonged sickness, to present it once more to the public, in an improved state, and under circumstances that must ensure its perusal by the great body of the reading population of these countries. It is a book truly matchless in its objects, in its plan, and in its execution; and we are certain that those members of our profession, who duly appreciate the great object of all medical science,—the improvement of human health,—can have no greater gratification, and can do few better actions, than using their influence to cause it to be read and studied by their lay friends, high and low, rich and poor. The sale of the former editions has been enormous both in this country and America; and when we consider that the present enlarged edition is sold at one third the price of the former, viz., *half-a-crown*, it may safely be calculated that the future diffusion of the work will be somewhat proportionate to its easier acquisition. The following extract from the preface commemorates a most important step in the progress of human civilization, which must have attracted the attention of every observant physician.

"Ten years ago, the proposition that physiology should constitute a part of general education, was received by most persons with ridicule or doubt, and by very many with absolute disgust. Of late, on the contrary, opinion has been almost unanimous in its favour, and allusions to the doctrines and usefulness of physiology meet us on every side. In accordance with this, its laws are now frequently referred to by men of philosophic minds, as the standards by which every proposition for the physical, social, or moral improvement of man ought to be tested. This happy change is likely to be progressive, because it has arisen from the gradual diffusion of sounder ideas, and from the preference instinctively felt for useful truths, when once clearly presented to the understanding." (Preface, p. vii.)

